# A Multi‐enzyme Cascade for the Biosynthesis of AICA Ribonucleoside Di‐ and Triphosphate

**DOI:** 10.1002/cbic.202100596

**Published:** 2021-12-16

**Authors:** Lobna Eltoukhy, Christoph Loderer

**Affiliations:** ^1^ Chair of Molecular Biotechnology Institute for Microbiology Technische Universität Dresden Zellescher Weg 20b 01217 Dresden Germany

**Keywords:** adenine phosphoribosyltransferase, enzymatic nucleotide synthesis, enzyme cascades, nucleotide analogues, polyphosphate kinases

## Abstract

AICA (5′‐aminoimidazole‐4‐carboxamide) ribonucleotides with different phosphorylation levels are the pharmaceutically active metabolites of AICA nucleoside‐based drugs. The chemical synthesis of AICA ribonucleotides with defined phosphorylation is challenging and expensive. In this study, we describe two enzymatic cascades to synthesize AICA derivatives with defined phosphorylation levels from the corresponding nucleobase and the co‐substrate phosphoribosyl pyrophosphate. The cascades are composed of an adenine phosphoribosyltransferase from *Escherichia coli* (*Ec*APT) and different polyphosphate kinases: polyphosphate kinase from *Acinetobacter johnsonii* (*Aj*PPK), and polyphosphate kinase from *Meiothermus ruber* (*Mr*PPK). The role of the *Ec*APT is to bind the nucleobase to the sugar moiety, while the kinases are responsible for further phosphorylation of the nucleotide to produce the desired phosphorylated AICA ribonucleotide. The selected enzymes were characterized, and conditions were established for two enzymatic cascades. The diphosphorylated AICA ribonucleotide derivative ZDP, synthesized from the cascade *Ec*APT/*Aj*PPK, was produced with a conversion up to 91 %. The *Ec*APT*/Mr*PPK cascade yielded ZTP with conversion up to 65 % with ZDP as a side product.

## Graphical abstract

**The biosynthesis of AICA** (5′‐aminoimidazole‐4‐carboxamide) nucleotides, the pharmaceutically active metabolites of AICA based drugs, was explored in this study. In an enzymatic cascade, a phosphoribosyl transferase is combined with two different polyphosphate kinases to selectively produce AICA ribonucleoside di‐ or triphosphate, respectively.

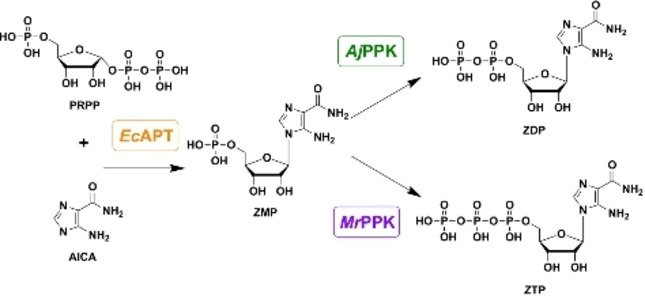

## Introduction

AICA or 5‐amino imidazole‐4‐carboxamide ribonucleoside is one of the ribonucleoside analogues, which is firmly established as a broad spectrum pharmaceutical in the clinical field. Based on its therapeutic relevance, AICA ribonucleoside has undergone extensive clinical studies over the last 25 years.^[1]^ In the early 90s, AICA ribonucleoside and its 5‐phosphorylated counterpart (ZMP) were first considered to be used as activators of the AMP‐activated protein kinase (AMPK).^[2]^ This activation is related to many beneficial metabolic events, such as the stimulation of glucose uptake and the inhibition of lipogenesis in the liver. The investigation revealed that AICA ribonucleoside could reduce cardiac death, myocardial infarction, and combined adverse cardiovascular outcomes.^[3]^ Many studies also approved that increased AMPK expression is related to inhibiting the proliferation of cancer cells in humans without affecting the viability and proliferation of non‐malignant cells.[^4^, ^5^] This finding led to using AICA ribonucleotides to demonstrate the effect of activation of AMPK in cells, an indication of its anti‐proliferative role in suppressing tumor formation.[^5^, ^6^] However, due to their polarity, the pharmaceutically active AICA mono‐, di‐ or triphosphates cannot pass the cell membrane.^[7]^ Therefore, the AICA ribonucleoside is administered as a prodrug that is phosphorylated in the target cells.

A reliable method for the synthesis of different AICA nucleotides is required to investigate the structure‐activity relationships, kinetics, and other metabolic consequences of nucleoside administration. Another possible application of AICA nucleotides is the preparation of protected nucleotides that can cross the biological barriers. Inside the cell, the protecting group is degraded, and the nucleotide analogue is released, then expresses its therapeutical potency.^[8]^

Typically, nucleotides such as AICA ribonucleotide are primarily produced by chemical methods like the Yoshikawa protocol or the Ludwig‐Eckstein method.^[9]^ However, it is tedious to use chemical techniques to synthesize nucleosides and their derivatives with high purity and convenient yield. Furthermore, it is a time‐consuming multi‐step method.^[10]^ As an alternative, the biosynthesis approach of nucleotides analogues by whole cells or isolated biocatalysts provides advantages compared to chemical processes, including moderate reaction conditions, high stereo, and regioselectivity.^[11]^ Moreover, the selection of suitable enzymes can significantly reduce production costs and transformation waste.

Since ribonucleotides are relatively complex molecules, they cannot be synthesized in a single enzyme reaction. Biocatalytic multi‐steps cascades composed of several enzymes have to be applied. The conditions of such enzyme cascades have to be optimized not only to achieve a suitable milieu for each individual reaction to avoid side reactions and inhibitory effects but also to obtain a balanced flow during the reaction cascade without the accumulation of intermediates.^[12]^

There are several biocatalytic approaches to synthesize non‐canonical ribonucleotides.^[11]^ One of the potential classes of enzymes to be used are nucleoside 2′‐deoxyribosyltransferases, which catalyze the exchange of the 2′‐deoxyribose moiety between a purine or pyrimidine 2′‐deoxynucleoside (donor) and a purine or pyrimidine base (acceptor).[^13^, ^14^] The combination of 2′‐deoxyribosyltransferases with other enzymes, such as nucleoside kinases,^[15]^ enables the synthesis of nucleoside‐5′‐mono, ‐di or triphosphates.^[16]^

Alternatively, transferases catalyze the addition of the nucleobase to activated ribose to obtain the nucleotide monophosphate.[^16^, ^17^] phosphoribosyltransferase catalyzes the reversible transfer of the 5‐phosphoribosyl group from 5‐phospho‐α‐D‐ribosyl‐1‐pyrophosphate (PRPP)^[17]^ to the nitrogenous base in the presence of Mg^2+^ and under pyrophosphate release.[^16^, ^17^, ^18^, ^19^] Further phosphorylation of the nucleotide can be achieved by employing kinases to obtain other phosphorylated derivatives.[^13^, ^19^] Examples are adenylate kinase, pyruvate kinase, polyphosphate kinase, and nucleoside diphosphate kinase.[^19^, ^20^] Polyphosphate kinases (PPK) catalyze the reversible phosphate transfer between a nucleotide and inorganic polyphosphate. Depending on the class of PPK, different phosphorylation levels of the nucleotide substrates can be phosphorylated. While PPK2‐II show a preference for phosphorylation of nucleoside monophosphate to diphosphates, PPK2‐III are able to form nucleoside tri‐ or even tetraphosphates.^[21]^ Furthermore, PPK enzymes have the capacity to phosphorylate a broad spectrum of nucleosides like guanosine, cytidine, and uridine, in addition to inosine and xanthosine.[^22^, ^23^]

This study aims to establish and characterize enzymatic cascades for the selective biosynthesis of the different phosphorylated derivatives of AICA ribonucleoside. First, an adenosine phosphoribosyltransferase (*Ec*APT) shall be applied to synthesize ZMP. Exploiting the phosphate specificities of polyphosphate kinases from different classes, selective cascades for the biosynthesis of ZDP and ZTP shall be established. The cascades will be characterized and evaluated for their suitability to produce these essential and valuable compounds.

## Results

**Figure 1:**
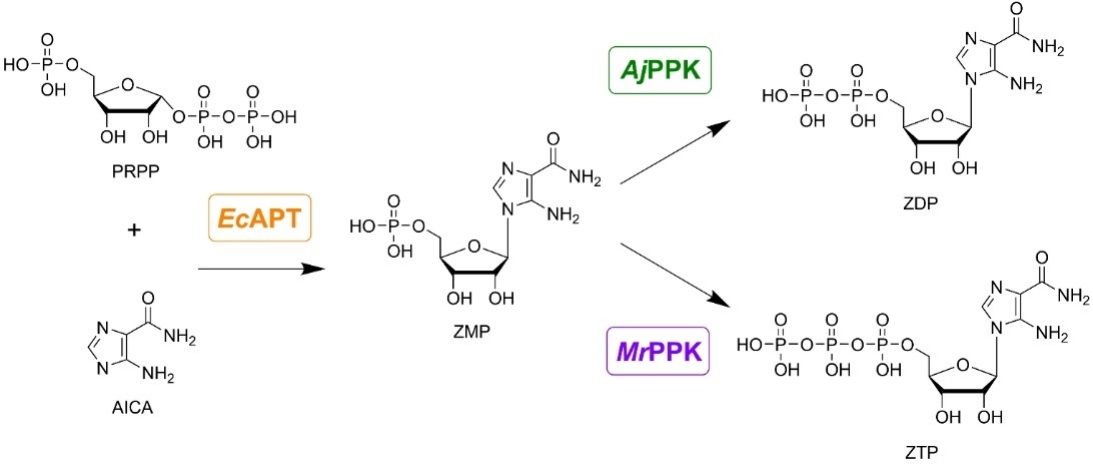
Desired cascades for synthesizing 5‐aminoimidazole‐4‐carboxamide ribonucleotide mono‐, di‐ and tri‐phosphate (ZMP, ZDP, and ZTP). The adenine phosphoribosyltransferase (*Ec*APT) catalyzes the coupling of the base AICA to the ribose moiety (PRPP) to form ZMP. A combination of the *Ec*APT with different polyphosphate kinases shall then be used to create cascades for the biosynthesis of ZDP (*Aj*PPK) and ZTP (*Mr*PPK).

Two specific cascades were conceived for the specific biosynthesis of ZDP and ZTP, respectively. Three enzymes were considered to set up the cascades: adenosine phosphoribosyl transferase (*Ec*APT) to couple the AICA base to the ribose moiety and two polyphosphate kinases with different phosphate specificities to produce the nucleoside di‐and triphosphate (*Aj*PPK and *Mr*PPK), respectively (Figure 1). All three enzymes were successfully produced in *E. coli* and purified by IMAC and desalting. The procedure yielded 78 mg, 45.6 mg, and 28.8 mg for *Ec*APT, *Aj*PPK, and *Mr*PPK, respectively, with good purities (Figure S1).

### Characterization of the single enzymes

**Figure 2:**
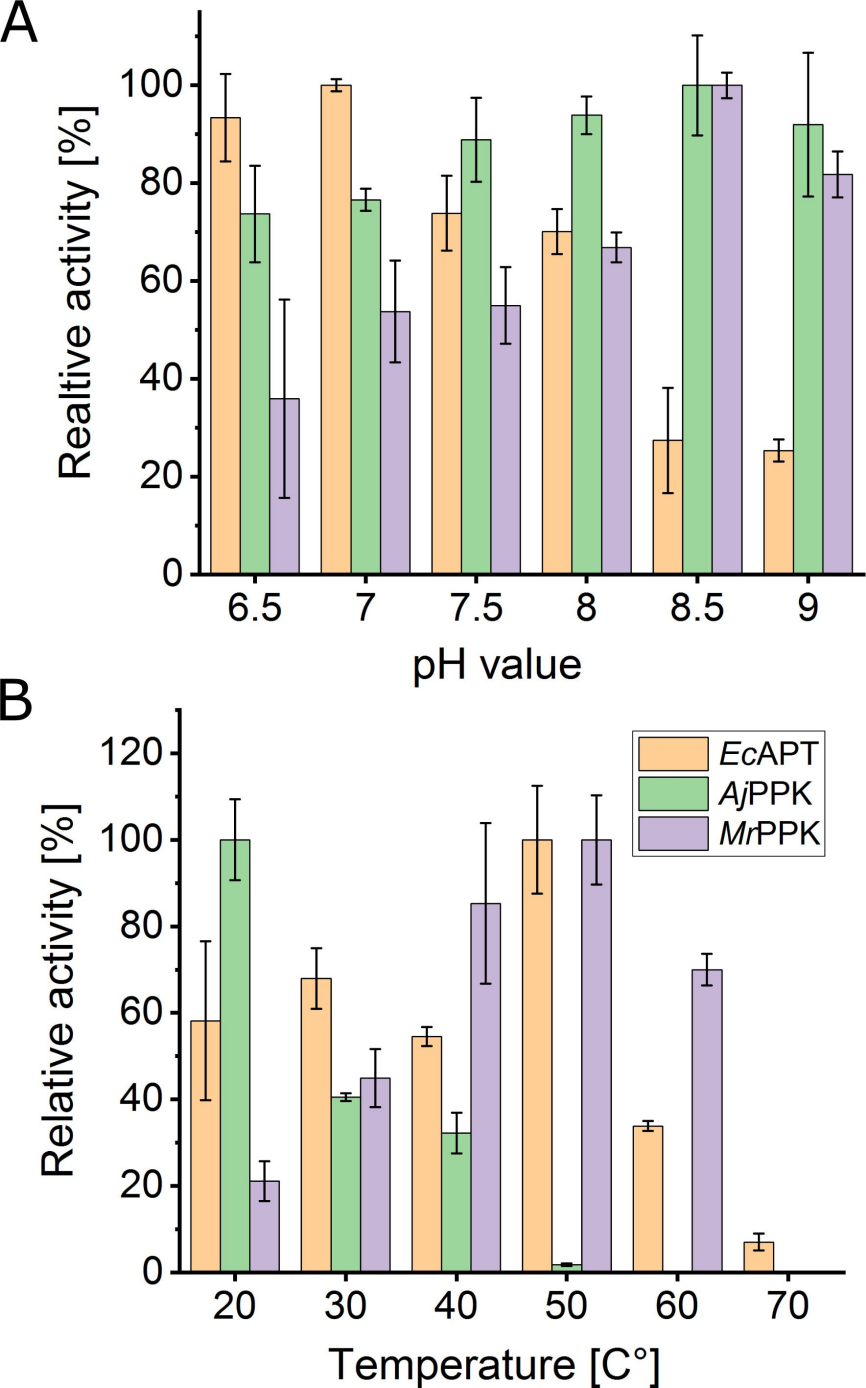
Influence of pH value (A) and reaction temperature (B) on the activity of the enzymes *Ec*APT, *Aj*PPK, and *Mr*PPK. For the pH experiments, the reaction temperature was kept constant at 40 °C for *Ec*APT and *Mr*PPK and at 30 °C for *Aj*PPK. All experiments were performed in tris‐HCl buffer, set to the respective pH Value. For the temperature experiments, the pH was maintained at 8.0 for all enzymes. The error bars indicate the standard deviation from the three independent replicates.

In preparation for the desired cascade reactions, the three enzymes were characterized for the conversion of the respective AICA ribonucleoside derivatives under different reaction conditions. Purified enzymes were examined for their activity in the pH range 6.5–9.0 and in the temperature range 20–80 °C. The pH profile showed the highest activity for *Ec*APT at pH=7.0, *Aj*PPK at pH=8.5, and *Mr*PPK at pH=8.5 (Figure 2A). Regarding the activity of the three enzymes, a pH of 8.0 was selected for all cascades. The incubation of the enzymes at different temperatures showed the highest activity of the *Ec*APT at 50 °C, while the optimal temperatures recorded for the *Aj*PPK, *Mr*PPK were 20 °C and 50 °C, respectively (Figure 2B).

To account for the enzyme stability in the longer cascade reaction, we selected 30 °C and 40 °C for the *Ec*APT/*Aj*PPK and *Ec*APT/*Mr*PPK cascades, respectively. For a possible combination of the three enzymes, 30 °C was the most appropriate selection because *Aj*PPK exhibited a limited temperature tolerance compared to the other enzymes.

The kinetic parameters towards AICA substrates were investigated for each biocatalyst. K_M_ and V_max_‐values were estimated by non‐linear regression of the experimental data to the Michaelis‐Menten equation (OriginPro 2019) (Table 1). Typical Michaelis‐Menten‐like behavior was observed for *Ec*APT and *Aj*PPK but not for *Mr*PPK (Figure S2). For the latter, reduced enzyme activity was observed at substrate concentrations above 14 mmol L^−1^. To get an estimate of the minimal K_M_‐value, we fitted the Michaelis‐Menten equation to the experimental data where no inhibition was obvious yet (Figure S2). It is, therefore, not a true K_M_‐value but an estimation of the minimal value. All enzymes exhibited high K_M_‐values in the 1–10 mmol L^−1^ range.

**Table 1:** Kinetic parameters of the selected enzymes *Ec*APT, *Aj*PPK, *Mr*PPK using different concentrations of the relevant substrate under conditions optimized for each cascade.

Enzyme	Substrate	V_max_ [U mg^−1^]	K_M_ [mmol L^−1^]
*Ec*APT	AICA	8.6±1.4	6.3±2.3
*Aj*PPK	ZMP	67±8.0	8.2±1.4
*Mr*PPK	ZMP	–	8.6±4.7^[a]^

[a] The displayed K_M_‐value for the *Mr*PPK is an estimate of the minimal value as described in the Supporting Information (Figure S2).

### One‐pot production of phosphorylated derivatives of AICA ribonucleoside

**Figure 3:**
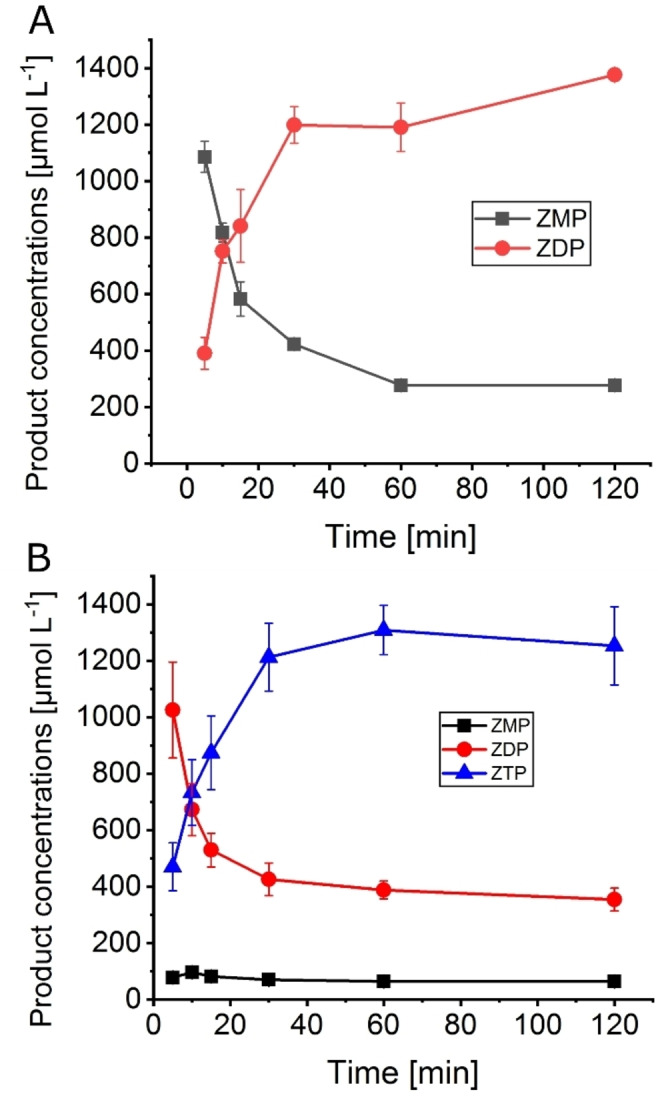
AICA nucleotide concentrations over the reaction time in the cascades *Ec*APT/*Aj*PPK (A) and *Ec*APT/*Mr*PPK (B). Enzyme concentrations: A: *Ec*APT 1 μmol L^−1^, *Aj*PPK 0.05 μmol L^−1^. B: *Ec*APT 1 μmol L^−1^, *Mr*PPK 10 μmol L^−1^. Substrate concentrations: 10 mmol L^−1^ AICA, 2 mmol L^−1^ PRPP. The error bars indicate the standard deviation from the three independent replicates.

According to the results of the characterization of the single enzymes, two enzyme cascades for the biosynthesis of phosphorylated derivatives of AICA ribonucleoside were set up, *Ec*APT/*Aj*PPK for ZDP and *Ec*APT/*Mr*PPK for ZTP synthesis. The substrate PRPP is the more valuable substrate, so its concentration was kept constant at 2 mmol L^−1^ for all cascade reactions. In the first set of experiments, the formation of the phosphorylated products was monitored for both of the cascades *Ec*APT/*Aj*PPK and *Ec*APT/*Mr*PPK over time. Over the course of the reaction, the highest concentration of ZDP was 1380 μmol L^−1^, corresponding to 69 % conversion along with 277 μmol L^−1^ of ZMP reached after 2 h for the *Ec*APT/*Aj*PPK cascade (Figure 3A). The highest concentration of ZTP was 1308 μmol L^−1^, equivalent to 65 % conversion for the *Ec*APT/*Mr*PPK cascade reached after 1 h (Figure 3). In addition, 388 μmol L^−1^ of ZDP and 65 μmol L^−1^ ZMP were detected at this point, as well.

LC‐MS analysis was applied to identify the formed compounds. The *Ec*APT/*Aj*PPK cascade delivered two chromatographic peaks at the retention times 3.47 and 6.01 min. Mass spectrometric analysis showed mass peaks at 337.1 and 417.02, corresponding to ZMP and ZDP, respectively. The *Ec*APT/*Mr*PPK cascade delivered two main chromatographic peaks at the retention times 6.04 and 7.67 min with mass peaks 417.02 and 496.98, corresponding to ZDP and ZTP (Figure S3).

**Figure 4:**
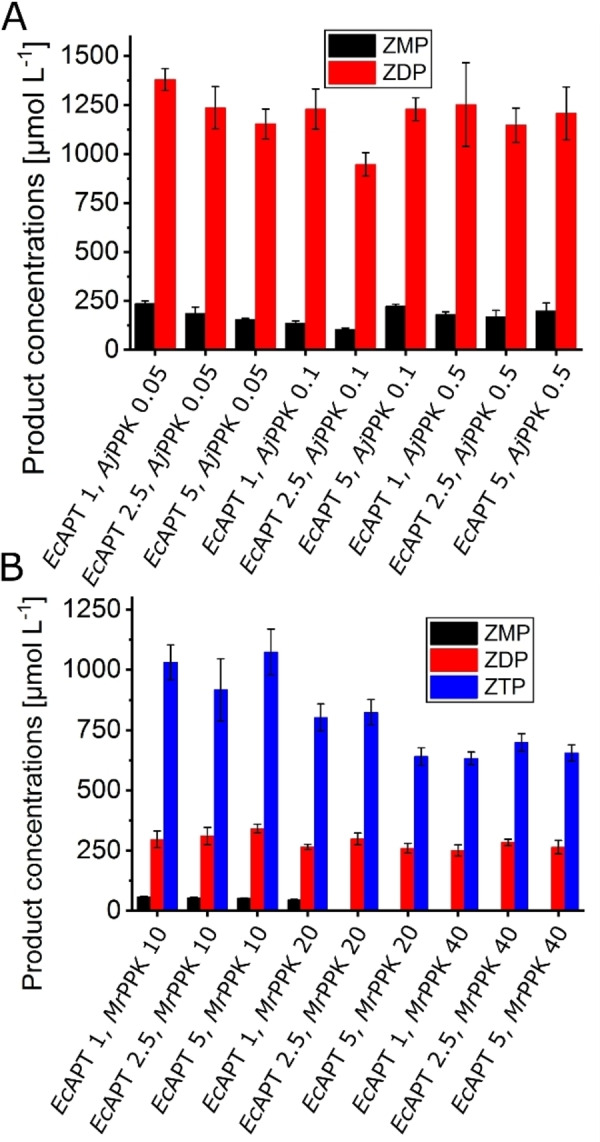
Effect of variation of enzyme concentrations. A. *Ec*APT/*Aj*PPK cascade with *Ec*APT concentrations between 1–5 μmol L^−1^ and *Aj*PPK concentrations between 0.05–0.5 μmol L^−1^. The reactions were performed for 2 h at 30 °C. B. *Ec*APT/*Mr*PPK cascade with *Ec*APT concentrations between 1–5 μmol L^−1^ and *Mr*PPK concentrations between 10–40 μmol L^−1^. Substrate concentrations: 10 mmol L^−1^ AICA, 2 mmol L^−1^ PRPP. The reactions were performed for 1 h at 40 °C. The error bars indicate the standard deviation from the three independent replicates.

The influence of the enzyme concentration on AICA nucleotide yield was investigated by the variation of both *Ec*APT and PPK concentrations. For the *Ec*APT/*Aj*PPK cascade, neither the variation of *Ec*APT (1–5 μmol L^−1^) nor *Aj*PPK (0.05–0.5 μmol L^−1^) resulted in improved ZDP formation (Figure 4). The best ZDP outcome was 1379 μmol L^−1^, almost 69 % conversion with a side formation of 236 μmol L^−1^ ZMP with only 1 μmol L^−1^ of the *Ec*APT and 0.05 μmol L^−1^ of the *Aj*PPK. In the *Ec*APT/*Mr*PPK cascade, the variation of the enzyme concentration of *Ec*APT (1–5 μmol L^−1^) and *Mr*PPK (10–40 μmol L^−1^) did not enhance the ZTP yield, either. However, with increasing *Mr*PPK concentration to 20 μmol L^−1^, another phosphorylated product started to form in lower concentrations. LC‐MS analysis indicates the formation of the tetra phosphorylated AICA nucleotide ZP_4_ (Figure S3B). The highest obtained yield from this cascade was 1073 μmol L^−1^ ZTP corresponding to 53.7 % conversion, besides 341 μmol L^−1^ of ZDP and 53 μmol L^−1^ of ZMP, in the presence of 5 μmol L^−1^ of the *Ec*APT and 10 μmol L^−1^ of the *Mr*PPK.

**Figure 5:**
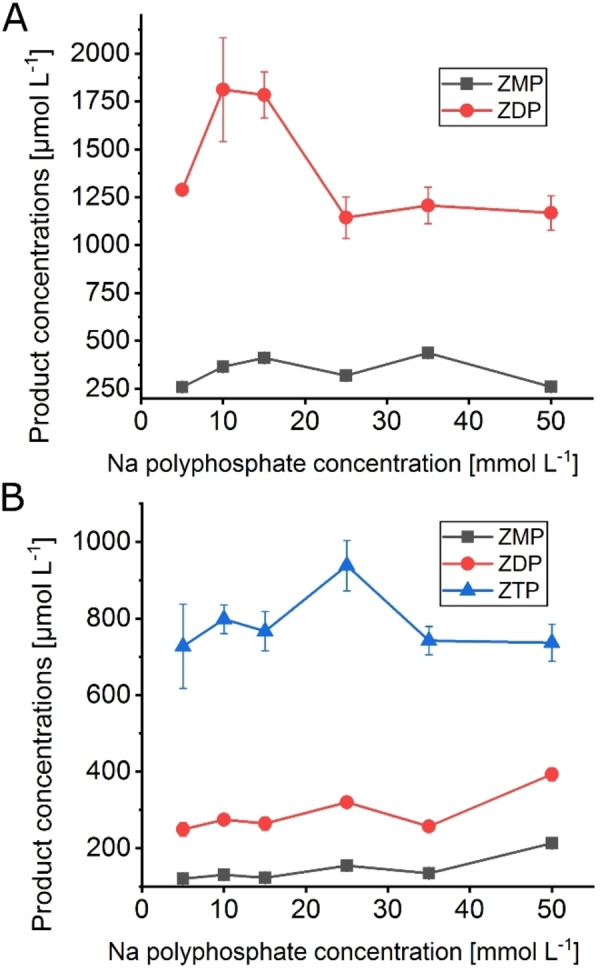
Effect of sodium polyphosphate concentrations (5–50 mmol L^−1^) on the cascades *Ec*APT/*Aj*PPK (A) and *Ec*APT/*Mr*PPK (B). Enzyme concentrations: A: *Ec*APT1, *Mr*PPK 0.1 μmol L^−1^; B: *Ec*APT 1, *Mr*PPK 10 μmol L^−1^. Substrate concentrations: 10 mmol L^−1^ AICA, 2 mmol L^−1^ PRPP. The reactions were performed for 2 h at 30 °C (A) and 1 h at 40 °C (B), respectively. The error bars indicate the standard deviation from the three independent replicates.

The effect of the sodium polyphosphate concentration on AICA nucleotide yield was analyzed by using different concentrations from 5–50 mmol L^−1^ for each cascade (*Ec*APT/*Aj*PPK, *Ec*APT/*Mr*PPK). Increasing the inorganic phosphate donor to a higher concentration did not seem to influence the reaction for both kinases dramatically. The highest impact was observed for the *Ec*APT/*Aj*PPK cascade, where the concentration of ZDP increased to 1812 μmol L^−1^, in the presence of 10 mmol L^−1^ of the Na‐polyphosphate, which corresponds to 91 % conversion and 365 μmol L^−1^ ZMP (Figure 5A). For the *Ec*APT/*Mr*PPK cascade, the highest concentration of ZTP was 938 μmol L^−1^ with 25 mmol L^−1^ Na‐polyphosphate, which corresponds to 47 % conversion and as side products 320 μmol L^−1^ of ZDP, 155 μmol L^−1^ ZMP (Figure 5B). High concentrations of the inorganic polyphosphate seemed to be detrimental for both kinases but might be required for higher conversions. Therefore, we tried supplying the reaction mixture with the polyphosphate source during the reaction. However, several different feeding patterns did not enhance the formation of the product for the *Ec*APT/*Mr*PPK cascade (Figure S4A).

To further assess the limiting component of the cascade, the concentration of the substrate PRPP was varied. Three concentrations of the PRPP were tested (1, 2, 5 mmol L^−1^) with no significant change in the ratios of formed AICA nucleotides (Figure S4B).

**Figure 6:**
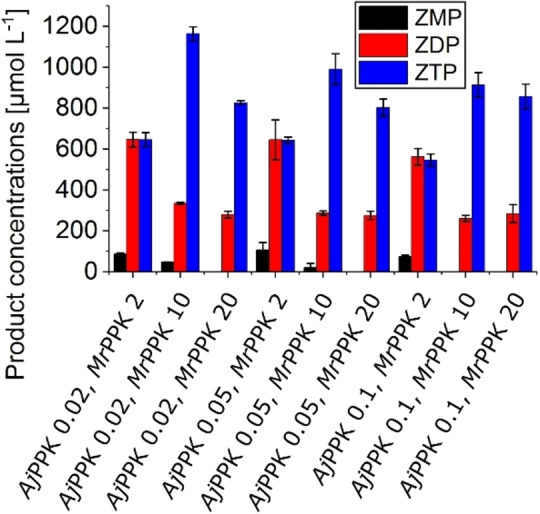
EcAPT/*Aj*PPK/*Mr*PPK cascade with *Ec*APT concentration 1 μmol L^−1^, *Aj*PPK concentrations between 0.02–0.1 μmol L^−1^, and *Mr*PPK concentrations between 2–20 μmol L^−1^ with 25 mmol L^−1^ sodium polyphosphate, 10 mmol L^−1^ AICA and 2 mmol L^−1^ PRPP. The reaction was performed for 2 h at 30 °C The error bars indicate the standard deviation from the three independent replicates.

While the *Ec*APT/*Aj*PPK cascade delivered ZDP with good selectivity and yield, the ZTP production by the second cascade leaves room for improvement. Therefore, we attempted the combination of all three aforementioned enzymes in one cascade (Figure 6). This cascade yielded 646 μmol L^−1^ ZTP in the presence of a low concentration of *Mr*PPK of 2 μmol L^−1^ and variation of the *Aj*PPK between 0.02, 0.05, and 0.1 μmol L^−1^. After increasing the *Mr*PPK concentration to 10 μmol L^−1^, the highest ZTP yield was 1164 μmol L^−1^ (58 % conversion), 335 μmol L^−1^ ZDP, and traces of ZMP.

## Discussion

The approach of combining an adenosine phosphoribosyl transferase with different polyphosphate kinases generally succeeded in producing different AICA ribonucleotides in various conversions up to 91 %. The three enzymes were extensively characterized in preparation for the cascade reactions. Finding the appropriate temperature and pH is a critical factor for the multi‐enzymatic transformation process, and it is challenging to optimize the conditions for each cascade. While both *Ec*APT and *Mr*PPK showed high activities up until 50 °C, *Aj*PPK exhibits lower temperature tolerance. Therefore, 30 °C was used as a compromise for the cascades that include *Aj*PPK. The *Ec*APT showed maximum relative activity at pH=7.0, while both kinases worked better at higher pH values, leaving a value of 8.0 as a compromise.

Another crucial factor is the kinetics of each enzyme. With 6.2 mmol L^−1^, the *Ec*APT shows a 50‐fold higher K_M_ value compared to the natural substrate adenine.^[24]^ Likewise, *Mr*PPK and *Aj*PPK showed a reduced affinity for the substrate ZMP in comparison to the natural substrate. The K_M_ for the *Aj*PPK is 8.2 mmol L^−1^ for ZMP compared to the published value for AMP, 0.27 mmol L^−1^.[^24^, ^25^] The same is true for the minimal K_M_‐value estimated for the *Mr*PPK. Since AICA nucleosides and nucleotides are not the natural substrates of these enzymes, lower binding affinities are to be expected. Still, the K_M_‐values are higher than the applied substrate concentrations in the cascades, limiting the efficiency of the biocatalysts.

Despite minor differences in pH and temperature preferences, functional cascades could be set up with both *Ec*APT/*Aj*PPK and *Ec*APT/*Mr*PPK. The highest conversions observed for the *Ec*APT/*Aj*PPK cascade were 91 % after 2 h. For the *Ec*APT/*Mr*PPK cascade, the conversion was in the range of 50–60 % ZTP and 15 % ZDP in 1 h. Also, traces of ZMP and ZP_4_ were detected. The latter cascade achieved the conversion to the target product ZTP in a reasonable concentration but with limited selectivity. Two additional attempts were made to improve on that by feeding the reaction with the inorganic polyphosphate and varying the PRPP concentration. Both tests did not significantly impact the ratio of the product ZTP and the side product ZDP. Hence, there might be a thermodynamic rather than a kinetic limitation for the formation of nucleotides with a higher phosphorylation level.

The application of biocatalytic strategies for synthesizing nucleotide analogues has already been successful before for other examples such as 2Cl‐deoxyadenosine triphosphate or Cladribine triphosphate, 5F‐UTP, and UDP. Cladribine triphosphate was yielded from a similar cascade using phosphoribosyl transferase followed by two consecutive phosphorylation and reduction with ribonucleotide reductase. The concentration of the end product was 800 μmol L^−1^, corresponding to 80 % conversion.^[24]^ 5F‐UTP was produced from a reaction comprised of ribokinase, phosphoribosyl pyrophosphate synthetase, and kinases. The final product was formed in a concentration of 800 μmol L^−1^, equivalent to 80 % conversion after 110 h.^[19]^ UDP was obtained by a different cascade, including uracil phosphoribosyltransferase and uridine monophosphate kinase. UDP was yielded with 58 % conversion (2.3 mmol L^−1^) in 90 min of reaction time.^[26]^ Fehlau et al. investigated the validity of a one‐pot cascade to produce natural and modified NTPs in the presence of ATP as a phosphate donor with a regeneration system. In a system that included three kinases, they succeeded in producing the fluoro analogues 5F‐CTP with 75 % conversion and 2F‐ATP with 27 % conversion of 1 mmol L^−1^ substrate after 19 h.^[27]^ In light of those previously described methods, the conversions and reaction yields in our comparably simple system are competitive with the existing methods. The method relies on the rather expensive PRPP as the initial substrate, but the use of sodium polyphosphate directly as a phosphate donor circumvents the use of ATP and its *in situ* regeneration.

The described cascades might not only be useful for the synthesis of AICA nucleotides alone. The used *Ec*APT shows some substrate promiscuity, but also other phosphoribosyltransferases might be used to catalyze the first step of the cascade for other nucleotides. The polyphosphate kinases in this study (*Aj*PPK, *Mr*PPK) can already accept a wide range of purine and pyrimidine substrates.[^21^, ^22^]

In conclusion, *Ec*APT/PPK‐based cascades are capable of the biosynthesis of AICA nucleotides with defined phosphorylation levels. Despite the described limitations in ZTP biosynthesis, the cascades grant fast and straightforward access to these essential compounds.

## Experimental Section

**Materials and methods**: If not stated differently, all the used chemicals were purchased from Sigma‐Aldrich (Steinheim, Germany) or Carl Roth (Karlsruhe, Germany). ZTP was purchased from Jena Bioscience (Jena, Germany).

**Plasmids and strains**: The recombinant expression of the three enzymes was performed in different strains of *Escherichia coli*. The applied genes were the *ecapt* from *E. coli* (Uniprot ID: P69503), the *ajppk* from *Acinetobacter johnsonii* (Uniprot ID: Q83XD3), and the *mrppk* from *Meiothermus ruber* (Uniprot ID: M9XB82). The expression vector pET28b(+) with an N‐terminal His‐tag, harboring each gene, was transformed in *E. coli* BL21(DE3) for *Ec*APT and *Aj*PPK and in *E. coli* SHuffle T7 Express for *Mr*PPK.

**Enzyme expression and purification**: For recombinant gene expression, pre‐cultures were inoculated with the respective strains and incubated overnight at 37 °C and 180 RPM. Main cultures, each containing 800 ml LB medium and 30 μg ml^−1^ kanamycin, were inoculated to an OD_600_ of 0.07 for the BL21(DE3) and 0.15 SHuffle T7 Express and incubated to an OD_600_ of 1.0 at 37 °C and 130 RPM. Main cultures were induced with IPTG to a final concentration of 0.1 mmol L^−1^, and expression proceeded at 37 °C for 16 h (*Ec*APT) or at 20 °C for 22 h (*Aj*PPK, *Mr*PPK). The cells were harvested by centrifugation (25 min, 4 °C, 4700 g) and resuspended in His‐wash buffer (50 mmol L^−1^ tris, 300 mmol L^−1^ NaCl, 10 mmol L^−1^ imidazole, pH=8.0). Crude extracts were produced by French press lysis of the cell suspensions three times at 10 MPa (Sim Aminco, Urbana, IL, USA). The resulting cell lysate was then centrifuged at 20.000 g for 45 min and filtered through a 0.22 μm filter (Millipore). The clear lysate was loaded on HisTrap™ FF crude 5 ml column (GE Healthcare, Chicago, IL, USA) pre‐equilibrated with the His‐wash buffer. The column was washed with three times the column volume. The bound proteins were eluted in His‐elution buffer (50 mmol L^−1^ tris, 300 mmol L^−1^ NaCl, 500 mmol L^−1^ imidazole, pH=8.0). Imidazole was removed from the eluted proteins via a HiPrepTM 26/10 desalting column (GE Healthcare, Chicago, Illinois, USA) in desalting buffer (50 mmol L^−1^ tris, 300 mmol L^−1^ NaCl, pH=8.0). Before storage at −80 °C, the purified desalted enzymes were supplemented with glycerol to a final concentration of 20 % (v/v), aliquoted, and frozen in liquid nitrogen. The concentrations of the purified enzymes were determined using the nanodrop via the 260/280 nm method. An SDS‐PAGE of the purified enzymes is shown in the Supplementary Data (Figure S1).

**Enzyme activity assays**: The standard activity assay for each of the three enzymes was conducted under the same general conditions. The typical reaction mixture was composed of 50 mmol L^−1^ tris buffer and 20 mmol L^−1^ MgCl_2_ adjusted to a pH value of 8.0 in a final volume of 50 μl. The experiments concerning the pH dependence of the reaction were performed in 50 mmol L^−1^ tris buffer set to the pH values 6.5 to 9.0. For the *Ec*APT reactions, the assay was performed by incubating 1 mmol L^−1^ AICA, 3 mmol L^−1^ phosphoribosyl pyrophosphate (PRPP), and 0.1 μmol L^−1^ enzyme at 40 °C. The PPK reactions were conducted using 2 mmol L^−1^ ZMP as substrate, 5 mmol L^−1^ sodium polyphosphate, where the concentration was calculated based on the molecular weight of 101.96 g mol^−1^ for a single phosphate monomer. The standard enzyme concentrations and reaction temperatures for the PPKs were 0.1 μmol L^−1^ for *Mr*PPK at 40 °C, and 0.01 μmol L^−1^ for *Aj*PPK at 30 °C. The assay was started with the addition of the respective enzyme to the reaction mixture. The enzymatic reactions were stopped by adding 50 μl of the reaction mixture to 50 μl of 100 % methanol, vortexing, and heating for 10 minutes at 70 °C. Afterward, the reaction mixture was diluted with 200 μl water and centrifuged for 10 minutes at 17.000 g and 4 °C. All assays were carried out in three independent replicates, and the quantitative analysis of the concentration of the formed products over time was determined by HPLC as described below.

**Enzyme cascade reactions**: The cascade reactions for the *Ec*APT/*Aj*PPK were performed in 50 mmol L^−1^ tris, 20 mmol L^−1^ MgCl_2_, 25 mmol L^−1^ sodium polyphosphate, 10 mmol L^−1^ AICA, 2 mmol L^−1^ PRPP, adjusted to a pH of 8.0 in a final volume of 50 μl at 30 °C. The same reaction mixture was used for the *Ec*APT/*Mr*PPK cascade. However, the temperature was elevated to 40 °C. The cascade combining the three enzymes was composed of the same mixture as for *Ec*APT/*Aj*PPK and incubated at 30 °C. The enzyme concentrations were varied in the cascades with the following standard concentrations *Ec*APT 1 μmol L^−1^, *Aj*PPK 0.05 μmol L^−1^, and *Mr*PPK 10 μmol L^−1^. Sodium polyphosphate concentrations were varied between 5 and 50 μmol L^−1^ with 10 and 25 mmol L^−1^ as standard concentrations for *Ec*APT/*Aj*PPK and *Ec*APT/*Mr*PPK, respectively. The reaction started by adding the respective enzymatic mixture, and samples were taken throughout the reaction time. The reactions were stopped by adding the mixture to 50 μl of 100 % methanol, vortexing, and heating for 10 minutes at 70° C. After that, the reaction mixture was diluted with 200 μl volume water and centrifuged for 10 minutes at 17.000 g and 4 °C. Quantification was performed via HPLC as described below.

**HPLC‐analytics**: The quantitative analysis with HPLC was carried out on a Knauer Azura®‐HPLC (Knauer, Berlin, Germany) with a Eurosphere II 100‐5C18 column (Knauer, Berlin, Germany). The analytics for ZMP, ZDP, and ZTP were performed at flow rate of 0.5 ml min^−1^ with the following buffers as mobile phase: buffer A: 50 mmol L^−1^ KP_i_‐Buffer (pH=7.0), 10 mmol L^−1^ tetrabutylammonium hydroxide (TBAH), and 10 % (v/v) methanol; buffer B: 50 mmol L^−1^ KP_i_‐Buffer (pH=7.0), 10 mmol L^−1^ TBAH, and 30 %(v/v) methanol. The buffers were applied in the following elution profile: 0 min 60 % A, 8 min 0 % A, 18 min 0 % A, 19 min 60 % A, 22 min 60 % A. The retention times of the measured eluted components are 3.3 min (AICA), 5.2 min (ZMP), 8.7 min (ZDP), 11.3 min (ZTP). Retention time definition and calibration were performed with analytical standards for each compound. Analytical standards were only available for AICA and ZMP, ZTP. The calibration for AICA, ZMP, and ZTP was carried out using analytical standards. The concentrations of other intermediates were estimated based on the calibration for ZMP and ZTP. Other intermediates or products were identified by mass spectrometry on Thermo Fisher Q Exactive LC‐MS (Thermo Fisher Scientific, Waltham, MA, USA) (Details in the Supplementary Data).

## Conflict of interest

The authors declare that they have no conflicts of interest with the contents of this article.

1

## Supporting information

As a service to our authors and readers, this journal provides supporting information supplied by the authors. Such materials are peer reviewed and may be re‐organized for online delivery, but are not copy‐edited or typeset. Technical support issues arising from supporting information (other than missing files) should be addressed to the authors.

Supporting Information

## Data Availability

The data that support the findings of this study are available from the corresponding author upon reasonable request.
