# Supplementary material for: A Multi‐enzyme Cascade for the Biosynthesis of AICA Ribonucleoside Di‐ and Triphosphate
Source: Chembiochem. 2021 Dec 16;23(3):e202100596. doi: 10.1002/cbic.202100596 (PMC9299608; doi:10.1002/cbic.202100596)
Supplement: Supplementary file 1 — Supporting Information [file CBIC-23-0-s001.pdf]

ChemBioChem

Supporting Information

A Multi-enzyme Cascade for the Biosynthesis of AICA Ribonucleoside Di- and Triphosphate

Lobna Eltoukhy and Christoph Loderer*

SDS Page of purified enzymes

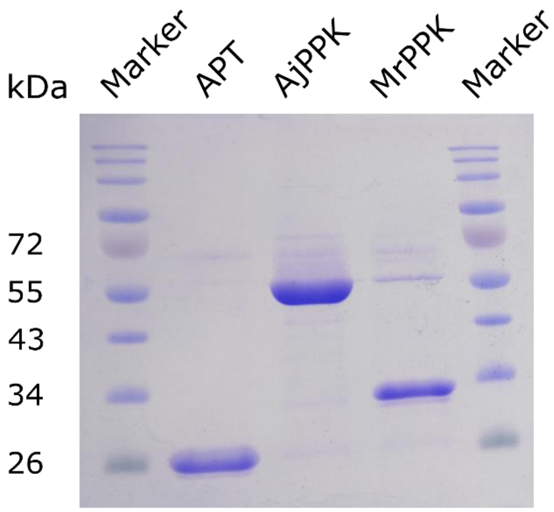

Figure S1: Purified and desalted samples loaded on 12% SDS-PAGE(M) wide molecular weight standard protein (10-250 kDa). The gel was stained with Coomassie Brilliant Blue.

Raw data – Enzyme kinetics

Kinetic parameters were estimated by non-linear regression of the experimental data to the original Michaelis-Menten equation. While APT and AjPPK showed Michelis-Menten like behavior (Figure S2 A,B), MrPPK exhibited reduced enzyme activity at higher ZMP concentrations (Figure S2 C). To estimate a minimal K_M-value, a regression was performed with the data up until a substrate concentration of 14 mmol L^{-1}, where no inhibition was obvious, yet (Figure S2 D). However, the values derived from this fit cannot be interpreted as K_M and V_{max} proper, but only as lower boundary for the true KM value of the enzyme for this substrate.

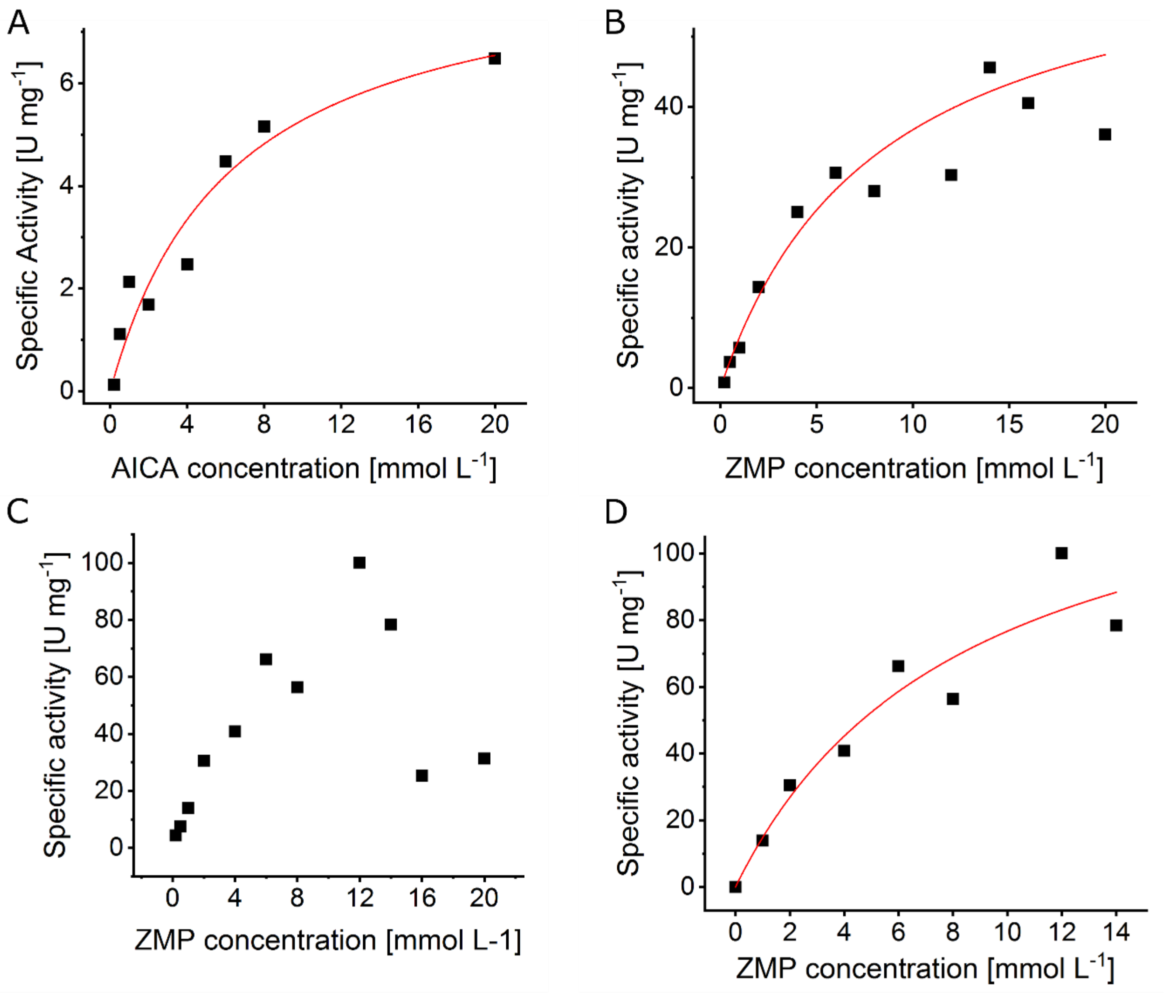

Figure S2: Non-linear regression of the Michelis-Menten equation (red) to the experimental data (black) for APT (A), AjPPK (B) and MrPPK (D). The results over the full concentration range for MrPPK are shown in C.

LC-MS method for identification of reaction products and intermediates

HPLC separation was performed on a Thermo vanquish-HPLC (Thermo Fisher Scientific, Waltham, MA, USA) with a ZIC®-cHILIC 3 µm, 100 Å column (Merck Millipore, Burlington, MA, USA). Samples prepared for the aforementioned HPLC method were diluted with one volume of acetonitrile. Separation was performed at a flow rate of 0.3 mL min^{-1} with the following eluents: (A) 10 mmol L^{-1} ammonium acetate buffer (pH = 6.8) and 75% (v/v) acetonitrile; (B) 10 mmol L^{-1} ammonium acetate buffer (pH = 6.8) and 0% (v/v) acetonitrile. The following elution profile was used: 0 min 100% A, 10 min 70% A, 12 min 25% A, 13 min 25% A, 13.5 min 100% A, 16 min 100% A.

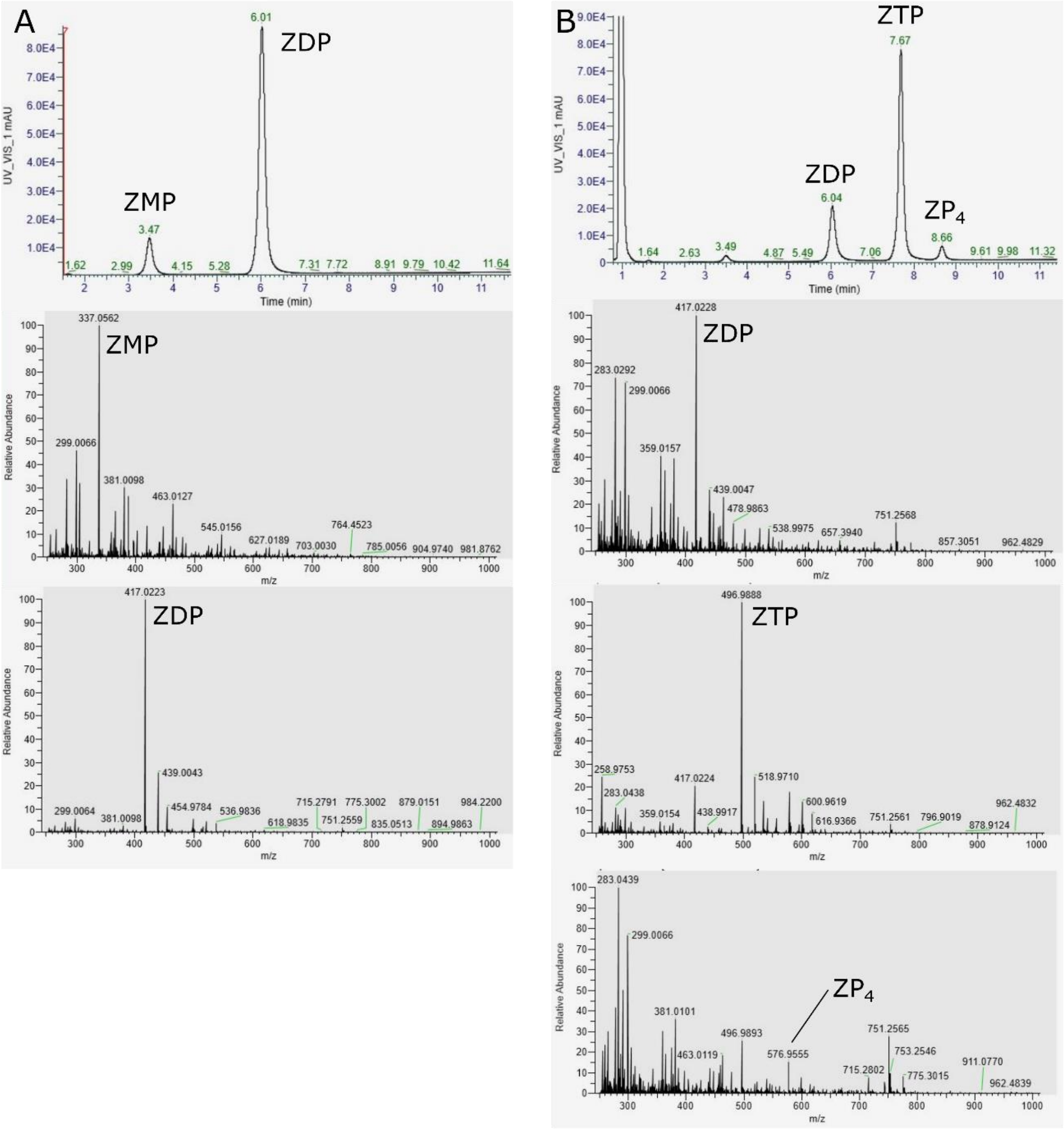

Figure S3: LC-MS results for the APT/AjPPK (A) and APT/MrPPK (B) cascades. For each chromatographic peak in the top picture, the mass spectra are shown below.

Mass spectrometric analysis was performed on a coupled Thermo Q Exactive Mass spectrometer (Thermo Fisher Scientific, Waltham, MA, USA). The analysis was performed with the following scan parameters: Polarity: Positive, AGC target: 3e6, maximum IT: 200 ms; scan range: 250 to 1000 m/z. Resolution 70,000. The electron spray settings were spray voltage: 4 kV; capillary temperature 320 °C; sheath gas flow rate: 25 mL min^{-1}; AUX gas flow rate: 10 mL min^{-1}; S-lens RF level: 55.

Cascade – Polyphosphate and PRPP experiments

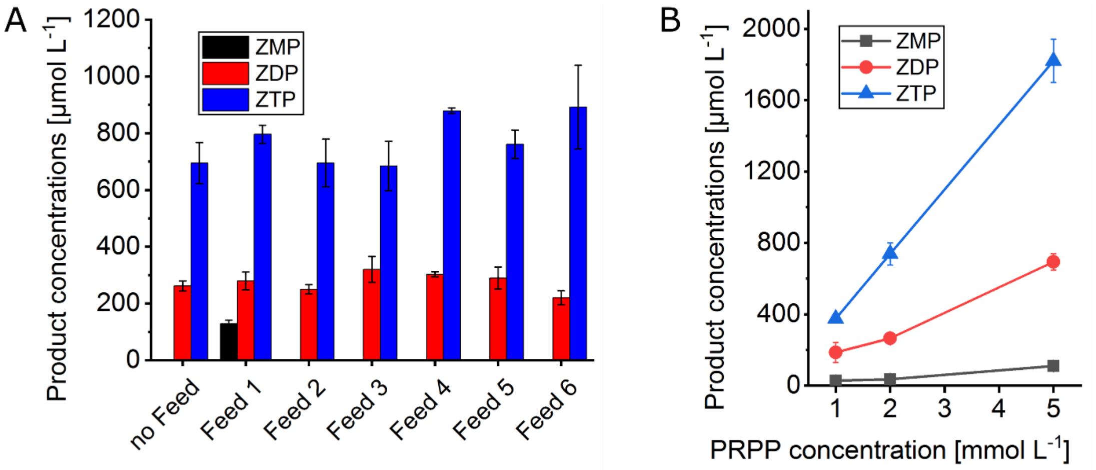

Figure S4: AICA nucleotide concentrations in the APT/MrPPK cascade with different polyphosphate feed strategies (A) and with varying PRPP concentrations (B). The Feed strategies are given in Table S1.

Table S1: Feed strategies for the polyphosphate feed in the APT/MrPPK cascade.

	Added polyP$_i$ [mmol L^{-1}]	*Number of additions*	*Time interval [min]*
No feed	0	0	0
Feed 1	10	2	15
Feed 2	10	4	15
Feed 3	25	2	15
Feed 4	25	4	15
Feed 5	35	2	15
Feed 6	35	4	15
